# Effectiveness of Combined Thrombolysis and Mild Hypothermia Therapy in Acute Cerebral Infarction: A Meta-Analysis

**DOI:** 10.1155/2022/4044826

**Published:** 2022-04-16

**Authors:** Lin Guo, Huaien Bu, Maojuan Guo, Yue Zhang, Qun Yu, Bin Yu, Xijuan Jiang, Lin Yang

**Affiliations:** ^1^School of Integrative Medicine, Tianjin University of Traditional Chinese Medicine, 10 Poyanghu Road, West Area, Tuanbo New Town, Jinghai District, Tianjin 301617, China; ^2^College of Traditional Chinese Medicine, Tianjin University of Traditional Chinese Medicine, Tianjin 300193, China; ^3^International Exchanges Department and International Education College, Tianjin University of Traditional Chinese Medicine, 10 Poyanghu Road, West Area, Tuanbo New Town, Jinghai District, Tianjin 301617, China

## Abstract

**Objective:**

To evaluate the effectiveness and safety of thrombolytic therapy combined with mild hypothermia in patients with acute cerebral infarction (ACI), based on a meta-analysis of randomized controlled trials (RCTs).

**Methods:**

PubMed, EMBASE, Cochrane Library, and Chinese National Knowledge Infrastructure Database of Controlled Trials were systematically screened for randomized controlled trials (RCTs) of thrombolytic therapy combined with mild hypothermia in treating ACI from inception to January 2021. Participation and outcomes among intervention enrollees are as follows: P, participants (patients in ACI); I, interventions (thrombolysis in combination with mild hypothermia therapy); C, controls (thrombolysis merely); O, outcomes (main outcomes are the change of NIHSS, glutathione peroxidase, superoxide dismutase, malondialdehyde, inflammatory factor interleukin-1*β*, tumor necrosis factor-*α*, and adverse reaction). Following data extraction and quality assessment, a meta-analysis was performed using RevMan 5.3 software.

**Results:**

A total of 26 RCTs involving 2071 patients were included. Compared to thrombolysis alone, thrombolytic therapy combined with mild hypothermia leads to better therapeutic efficacy [RR = 1.23, 95% CI (1.16, 1.31)], NIHSS [MD = −2.02, 95% CI (−2.55, −1.49)], glutathione peroxidase [MD = 8.71, 95% CI (5.55, 11.87)], superoxide dismutase [MD = 16.52, 95% CI (12.31, 19.74)], malondialdehyde [MD = −1.86, 95% CI (−1.98, −1.75)], interleukin-1*β* [MD = −3.48, 95% CI (−4.88, −2.08)], tumor necrosis factor-*α* [MD = −0.46, 95% CI (−3.39, 2.48)], and adverse reaction [RR = 0.87, 95% CI (0.63, 1.20)].

**Conclusions:**

Thrombolytic therapy combined with mild hypothermia demonstrates a beneficial role in reducing brain nerve function impairment and inflammatory reactions in ACI subjects analysed in this meta-analysis.

## 1. Introduction 

Ischemic stroke, also known as cerebral infarction, is caused by deprivation of blood and oxygen supply due to thrombosis or thromboembolism. According to the statistics of China Clinical Research Center for Neurological Diseases and China Stroke Association, the number of people who died from cerebrovascular disease (CVD) in 2018 was as high as 1.57 million, which accounts for 80–90% of all stroke cases, and acute cerebral infarction (ACI) accounts for 70% of ischemic stroke [1]. ACI is one of the main causes of death and long-term disability in the world. In 2010, the number of patients with ACI in the world was 33 million. According to epidemiological speculation, it will increase to 77 million by 2030 [2–4]. Therefore, effective therapeutic intervention for ACI could reduce CVD morbidity and mortality. Thrombolysis using medication is usually the first choice to treat ACI, which restores impeded blood flow and protects adjacent brain tissue [5, 6]. Recombinant thrombolytic tissue plasminogen activator (rtPA) is the only drug licensed by the U.S. Food and Drug Administration (FDA) to treat ACI [7, 8]. RtPA activates plasminogen to plasmin, which dissolves thrombus to alleviate ischemia and hypoxia [9–11].

In thrombolytic therapy, the time window is a key determinant of brain metabolic rate and treatment effect [12, 13]. However, the time window of rtPA to treat ACI is within 4.5 hours. Most patients fail to reach maximum benefit because of missing the timely detection and timely intervention [9]. Therefore, it is necessary to investigate the therapy for ACI. Mild hypothermia is of neuroprotective effect by attenuating secondary injury after primary neurological insult [14]. Mild hypothermia treatment can slow down brain metabolism, reduce energy and oxygen demand, and reduce the production of oxygen-free radicals by maintaining its core temperature between 33°C and 34°C [15]. As a result, cerebral infarction, brain edema, and intracranial hypertension are alleviated [16].

In terms of efficacy and safety, thrombolytic therapy combined with mild hypothermia is a promising method to treat patients with ACI compared to thrombolysis alone [16–18]. However, this combined application has not been widely used in clinical practice yet. To better understand the efficacy and safety of this combination therapy, we performed a meta-analysis of existing randomized controlled trials (RCTs).

## 2. Materials and Methods

### 2.1. Search Strategy

The PubMed, EMBASE, Cochrane Library, and Chinese National Knowledge Infrastructure Database of Controlled Trials were systematically searched for randomized controlled trials (RCTs) of thrombolytic therapy combined with mild hypothermia in treating ACI from the inception of the databases to January 2021. Using key words including “stoke,” “brain infarction,” “ischemic stroke,” “cerebral infarction,” “brain embolism,” “cerebrovascular disorders,” “hypothermia,” “mild hypothermia,” “mild hypothermia therapy,” “TH,” “alteplase,” “rtPA,” and “Tissue Plasminogen Activator.”

### 2.2. Search Strategy in PubMed

“Brain Infarction”[Mesh]
“Acute Cerebral Infarction”[Title/Abstract] OR “Acute Stoke”[Title/Abstract] OR “Acute Brain Embolism”[Title/Abstract] OR “Acute Ischemic Stroke”[Title/Abstract]
(1) OR (2)
“hypothermia”[Title/Abstract] OR “mild hypothermia”[Title/Abstract] OR “mild hypothermia therapy”[Title/Abstract] OR “TH”[Title/Abstract]
“Tissue Plasminogen Activator”[Title/Abstract] OR “alteplase”[Title/Abstract] OR “rtPA”[Title/Abstract]
(3) AND (4) AND (5)

### 2.3. Study Selection

The titles and the abstracts of all publications obtained from the search strategies were screened by two reviewers. The eligibility criteria follow the PICOS framework [19].

#### 2.3.1. Participants

The participants were adults aged 18 years or older, all ACI patients included in the study were confirmed by cranial computed tomography and/or magnetic resonance imaging, and cerebral hemorrhage had been excluded [20].

#### 2.3.2. Interventions

Three approaches, including thrombolytic therapy, mild hypothermia treatment, and alone or in combination, were reviewed. Moreover, therapeutic hypothermia's information about the depth, duration, and rewarming speed had to be available for the study included.

#### 2.3.3. Controls

Only thrombolytic agent (rtPA) and routine treatment were included in the treatment of ACI.

#### 2.3.4. Outcomes

Clinical treatments including clinical efficacy and clinical outcome according to NIHSS are as follows: cure, NIHSS decreased by 91–100%; significant effective, NIHSS decreased by 46–90%; effective, NIHSS decreased by 18–45%; ineffective, NIHSS score decreased by 17% or less. The total clinical effective rate (%) = (cured cases + significant effective cases + effective cases)/total cases × 100%. There are some indexes for comparison between the control group and the intervention group, including clinic efficacy, NIHSS, superoxide dismutase (SOD), malondialdehyde (MDA), interleukin-1*β* (IL-1*β*), tumor necrosis factor-*α* (TNF-*α*), glutathione peroxidase (GSH-Px), and the adverse reactions.

The patient's family members gave informed consent and signed the informed consent form. Only RCTs were included.

### 2.4. Exclusion Criteria

Exclusion criteria were as follows: (1) duplicate article among databases; (2) nonclinical trials, such as animal experiments, pharmacology, and pharmacokinetics; (3) nonRCT research, such as literature review, expert experience, and mechanism elaboration; (4) ACI patients included in the study are complicated by other intracranial lesions, hypotension, uncorrected shock, cerebral hemorrhage, coagulation function, organ dysfunction, severe combined injury, or systemic failure, or patient had recent history of major surgery or cerebrovascular accident and literature that does not meet the requirements of this study; (5) patients with severe cardiac, hepatic, and renal insufficiency; (6) patients with abnormal coagulation function or recent use of anticoagulant drugs; (7) patients with intracranial aneurysm and tumor.

### 2.5. Quality Evaluation and Data Extraction

Two reviewers independently screened the titles and abstracts of search research articles to test whether they meet the review conditions. After meeting the criteria, the full text is read and evaluated according to the specified qualification criteria: baseline characteristics of the study population, interventions of therapeutic hypothermia (depth, duration, and rate of rewarming of therapeutic hypothermia), controls, and outcomes. If the reviewers have any differences, the third reviewer will solve the problem through discussion with them.

The methodological quality of the included studies was evaluated according to the quality evaluation criteria recommended in the Cochrane system evaluation manual [21, 22]: (1) a randomly assigned method, (2) allocation concealment, (3) use of blinding, (4) data integrity, (5) selectively reported results, and (6) the presence of bias. “Low risk” means low deviation risk, “high risk” means high deviation risk, and “unclear risk” means that the literature does not provide sufficient deviation evaluation information.

The information which we extracted were as follows: (1) bibliographic information; (2) demographic characteristics of ACI patients; (3) duration of thrombolytic therapy; (4) the number of ACI patients included in the study and the proportion of female patients; (5) therapeutic hypothermia (depth, duration, and rewarming speed); (6) outcomes of clinical treatments including clinical efficacy, NINSS, GSH-Px, SOD, MDA, IL-1*β*, TNF-*α*, and adverse reactions between two groups.

### 2.6. Statistical Analysis

The statistics analysis was performed using Review Manager 5.3 software (Cochrane). Risk ratio (RR) was used to analyse count data while mean difference (MD) was applied to analyse continuous variables. The chi-squared test and the I-squared statistic were used to appraise the heterogeneity. All studies used the random-effects model. The funnel plot discusses publication bias.

The standard deviation (SD) of baseline changes in the intervention groups was determined using the following equation, with *R*1 = 0.5 [21]:

(1) $$\begin{array}{c} \text{SD}\left(C\right)=\text{SD}\sqrt{\text{SD}{\left(B\right)}^{2}+\text{SD}{\left(F\right)}^{2}-2\times R1\times \text{SD}\left(B\right)\times \text{SD}\left(F\right).} \end{array}$$

Here, “SD (B)” is the standard deviation before intervention, while “SD (F)” means the standard deviation after intervention. *P* < 0.05 was considered statistically significant.

## 3. Results

### 3.1. Data Extraction and Assessment of Risk of Bias

**Figure 1:**
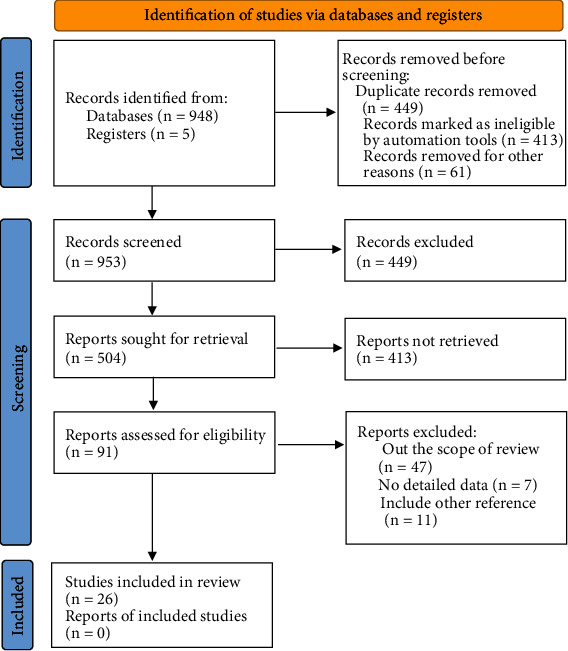
Flow chart of literature retrieval and selection.

Initially, 953 publications were identified, among which 140 articles are from Chinese National Knowledge Infrastructure database, 183 articles from VIP database, 186 articles from Wanfang database, 120 articles from Chinese Biomedical Literature Database, 39 articles from PubMed, 280 articles from EMBASE, and 5 from registration study. After reading the full text, the articles that did not meet the inclusion criteria were excluded, and 26 RCTs were finally included (Figure 1.

**Figure 2:**
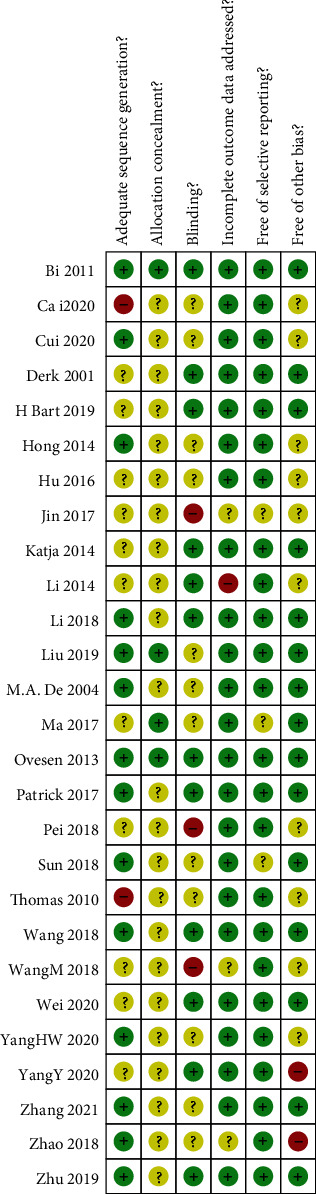
Details of risk of bias.

**Figure 3:**
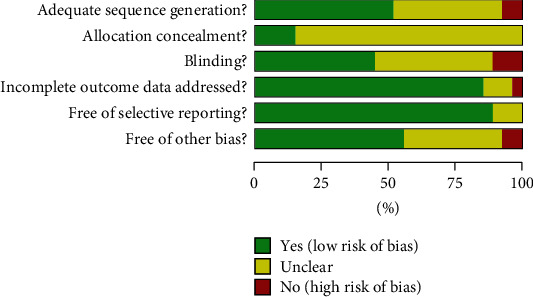
Risk of bias statistics: percentage of each risk of bias item across all included studies.

The quality assessment is shown in Figure 2 details of risk of bias are shown in Figure 3.

### 3.2. Patient Characteristics and Trial Design

In total, 2071 patients were included, among which 1058 subjects were in the treatment groups, whereas 1013 subjects were in the control groups. RtPA was selected as thrombolytic drug. Mild hypothermia treatment controls its core temperature between 33°C and 35°C. At the end of hypothermia therapy, most patients choose to rewarm. The details of RCTs are listed in Table 1 and the cooling characteristics of included studies are listed in Table 2.

### 3.3. Meta-Analysis Results

#### 3.3.1. Total Effective Rate

**Figure 4:**
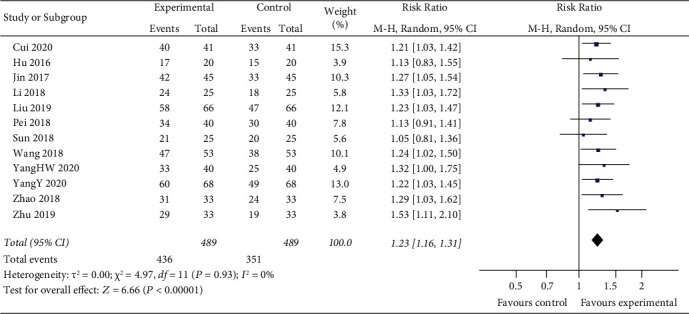
Effective rate of clinical efficacy rate between two groups.

Twelve studies demonstrated the clinical efficacy of the thrombolytic therapy combined with mild hypothermia used for ACI. The random-effects model is used for analysis, and the results showed that there was a highly significant statistical difference between the two groups (*z* = 6.66, *P* < 0.00001). The outcomes indicated the clinical efficacy rate in thrombolytic therapy combined with the mild hypothermia group was higher than that in the control group [RR = 1.23, 95% CI (1.16, 1.31)] (Figure 4.

#### 3.3.2. NIHSS Level

**Figure 5:**
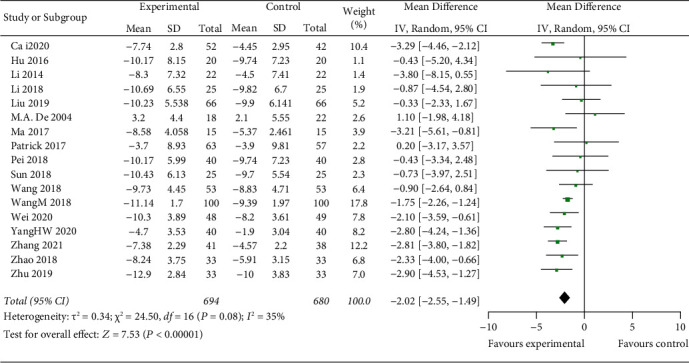
Compare change of NIHSS between two groups using meta-analysis.

Seventeen studies include NIHSS to assess ACI treatment. The random-effects model is used for analysis, and the results showed that there was a highly significant statistical difference between the two groups (*z* = 7.53, *P* < 0.00001). After treatment, the change of NIHSS in the experimental group was higher than that of the control group [MD = -2.02, 95% CI (−2.55, −1.49)] (Figure 5.

#### 3.3.3. GSH-Px Level

**Figure 6:**
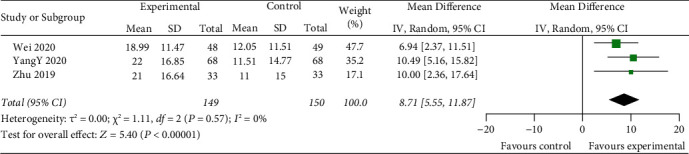
Compare change of serum GSH-Px levels between two groups.

Three studies include GSH-Px to appraise clinical outcomes of ACI treatment. The random-effects model is used for analysis, and the results showed that there was a highly significant statistical difference between the two groups (*z* = 5.40, *P* < 0.00001). After treatment, the change of serum GSH-Px in the experimental group was higher than that in the control group [MD = 8.71, 95% CI (5.55, 11.87)] (Figure 6).

#### 3.3.4. SOD Level

**Figure 7:**
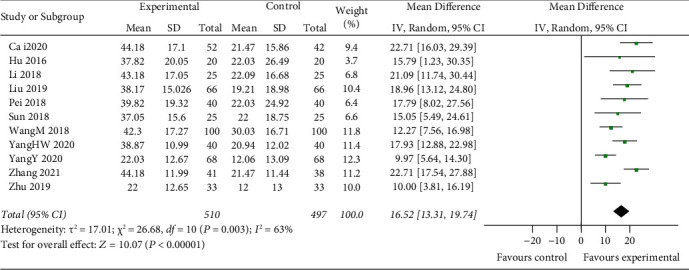
Compare change of serum SOD levels between two groups.

Eleven studies include SOD as a parameter of clinic. The random-effects model is used for analysis, and the results showed that there was a highly significant statistical difference between the two groups (*z* = 10.07, *P* < 0.00001). After treatment, the change of SOD in the experimental group was higher than that in the control group [MD = 16.52, 95% CI (13.31, 19.74)] (Figure 7).

#### 3.3.5. MDA Level

**Figure 8:**
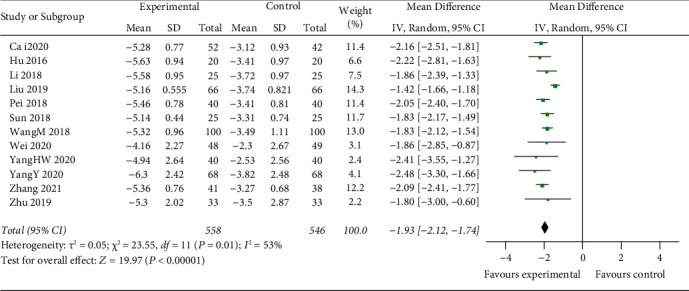
Compare change of serum MDA levels between two groups.

Twelve studies include MDA as the parameter of clinic. The random-effects model is used for analysis, and the results showed that there was a highly significant statistical difference between the two groups (*z* = 19.97, *P* < 0.00001). After treatment, the change of MDA in the experimental group was higher than that in the control group [MD = −1.86, 95% CI (−1.98, −1.75)] (Figure 8).

#### 3.3.6. IL-1*β* Level

**Figure 9:**
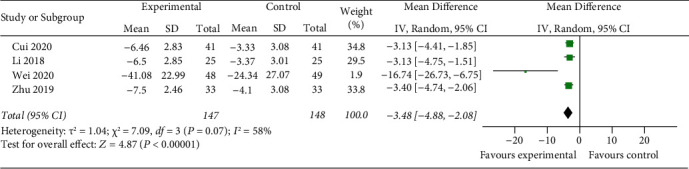
Compare change of serum IL-1*β* levels between two groups.

Four studies showed the changing of IL-1*β* before and after treatment in two groups. The random-effects model is used for analysis, and the results showed that there was a highly significant statistical difference between the two groups (*z* = 4.87, *P* < 0.00001). After treatment, the change of IL-1*β* in the experimental group was higher than that in the control group [MD = −3.48, 95% CI (−4.88, −2.08)] (Figure 9).

#### 3.3.7. TNF-*α* Level

**Figure 10:**
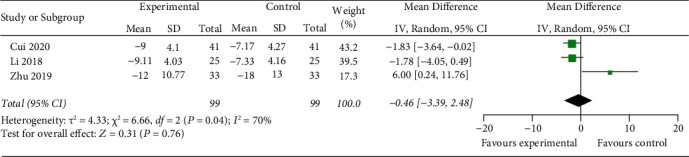
Compare change of serum TNF- *α* levels between two groups.

Three studies showed the changing of TNF-*α* before and after treatment in two groups. The random-effects model is used for analysis, and the results showed that there was no significant difference between the two groups (*z* = 0.31, *P*=0.76) [MD = −0.46, 95% CI (−3.39, 2.48)] (Figure 10).

#### 3.3.8. Adverse Reactions

**Figure 11:**
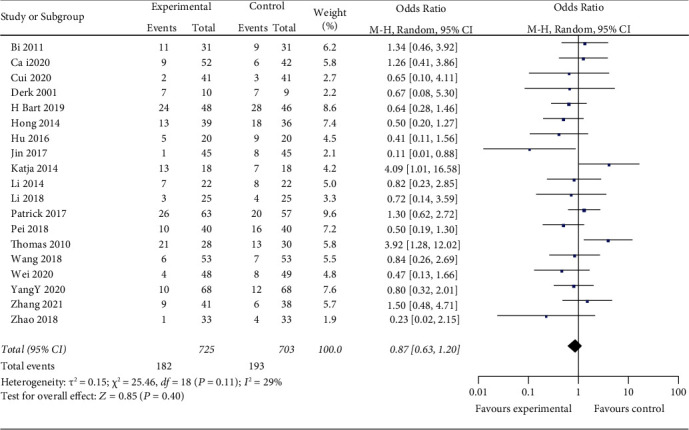
Adverse drug reaction between two groups.

Nineteen studies indicated adverse reactions between two groups. The random-effects model is used for analysis, and the results showed that there was no significant difference between the two groups (*z* = 0.85, *P*=0.40) [RR = 0.87, 95% CI (0.63, 1.20)] (Figure 11).

#### 3.3.9. Publication Bias

**Figure 12:**
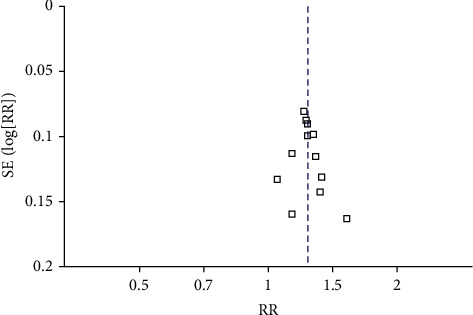
The forest plot of clinical efficiency.

Clinical efficacy rate was adopted as the outcome criteria to compare mild hypothermia combined with thrombolytic therapy in treating ACI. Funnel plot analysis was conducted based on these eleven studies included. The funnel plot is used to test the publication bias of clinical efficacy. According to the distribution of funnel plot, there was a certain publication bias (Figure 12).

## 4. Discussion

### 4.1. Major Findings

Cerebral infarction will ensue if the blood flow of ischemic brains is not restored promptly. A large number of dead cells release damage associate patterns and pathogen-related molecular patterns to activate the innate immune response, promote maturation or secretion of inflammatory cytokines, and further aggravate cerebral ischemic injury [48, 49]. Fibrin is an integral part of vascular thrombosis, which is degraded by plasmin that leads to the resolution of the thrombus. RtPA is a plasminogen activator that is widely used in the treatment of ACI and is the preferred drug for thrombolysis [5, 6]. In this analysis, rtPA is selected as the only thrombolytic drug to screen against literature. Moreover, NIHSS, whose high score indicates *n* neurological deficits, decreased significantly (*z* = 7.53, *P* < 0.00001) [MD = −2.02, 95% CI (−2.55, −1.49)], in thrombolytic therapy combined with mild hypothermia than thrombolysis alone. So, we speculated that thrombolytic therapy combined with mild hypothermia can highly reduce the mental injury of ACI patients.

IL-1*β* and TNF-*α* play a key role in the neuroimmune development of stroke by promoting neurotoxicity and the development of harmful inflammation after cerebral ischemia [50–53]. However, due to the limited number of studies on inflammatory indicators, the results reflect a better apparent effectiveness of IL-1*β* (*z* = 4.87, *P* < 0.00001) [MD = −3.48, 95% CI (−4.88, −2.08)]; the results do not reflect the more apparent effectiveness of TNF-*α* (*z* = 0.31, *P*=0.76) [MD = −0.46, 95% CI (−3.39, 2.48)]. Oxidative stress followed by releasing numerous oxygen-free radicals results in impairment of neurological function and mental state of patients. GSH-Px activity is an indicator of the oxidative stress response in ACI patients [54]. The level of SOD and MDA can indirectly reflect the antioxidant capacity of the body and the extent of nerve cell damage [54–56]. The effectiveness of thrombolysis combined with mild hypothermia for treating ACI was evaluated by oxidative stress indicators. The combination performs significantly better than rtPA alone: GSH-Px (*z* = 5.40, *P* < 0.00001) [MD = 8.71, 95% CI (5.55, 11.87)]; SOD (*z* = 10.07, *P* < 0.00001) [MD = 16.52, 95% CI (13.31, 19.74)]; MDA (*z* = 19.97, *P* < 0.00001) [MD = −1.86, 95% CI (−1.98,−1.75)]. Common adverse reactions of hypothermia treatment include pulmonary infection, urinary tract infection, venous thrombosis, intracranial hemorrhage, cerebral hernia, arrhythmia, upper gastrointestinal bleeding, gingival bleeding, and even death [57]. Only 11 RCTs in this study reported complications of hypothermia, and this study did not analyse independent diseases, which may have an impact on the results of meta-analysis.

Currently, there are two studies that assess the clinical application of adding mild hypothermia as a medication for thrombolysis to treat ACI [15, 58]. One study compares parameters of core temperature, duration and rewarming speed of therapeutic hypothermia, and occurrence of pneumonia as a complication of hypothermia. However, the thrombolytic drugs in the control group were unrestricted to rtPA. It was shown that hypothermia decreases the infarct area by 44% (95% confidence interval [CI], 40–47%) and the occurrence of pneumonia [RR = 3.30, 95% CI (1.48–7.34), *P*=0.003]. Whereas, there was no significant difference in mortality between different depths, duration, and rewarming speed, as well as the fatal intracranial hemorrhage and atrial fibrillation, upon addition of hypothermia treatment compared to thrombolysis alone. Andrea conducted another meta-analysis using the modified Rankin scale [mRS] to examine the disability of daily activity. It was demonstrated that mRS is significantly lower [RR = 1.17, 95% CI (0.93–1.46), *P*=0.02], while the cerebral infarction volume is also significantly smaller in the mild hypothermia combined with the rtPA treatment group compared with rtPA alone. Furthermore, the study conducted a more in-depth analysis of complications. It found that the incidence of complications was the highest in the highest within 2 hours. Among all hypothermia complications, cardiac complications were the highest, which was different from that in the prior study.

In this study, we selected studies that use the thrombolytic drug rtPA as the only control drug to treat ACI. RtPA combined with mild hypothermia was used to compare with the control group. Urokinase and streptokinase are also often used as thrombolytic agents to treat ACI. Their mechanism is also to mediate the transformation of plasminogen to plasmin, dissolve thrombus, and alleviate penumbra ischemia and hypoxia, so as to achieve the therapeutic effect [7, 59]. Urokinase and streptokinase combined with mild hypothermia in the treatment of ACI also have certain results, but the results of relevant studies are relatively limited and different from those included in this study, so we only included rtPA as a thrombolytic drug [60, 61]. The therapeutic effects were evaluated using NIHSS before and after treatment to exclude the influence of other possible thrombolytic drugs on the results. Among the 26 RCT studies that were selected against our inclusion criteria, demographic characteristics including gender, age, start time of thrombolysis, initial NIHSS, and details of mild hypothermia treatment including duration, target temperature, rewarming rate, and rewarming time of mild hypothermia treatment were explained. Compared with the existing analytical literature, our study includes NIHSS to evaluate the clinical effectiveness of thrombolysis-assisted mild hypothermia treatment.

### 4.2. Limitations

① The route of administration (intravenous injection or intra-arterial injection), dosage and administration time as well as cooling time and rewarming rate of hypothermia treatment varies between studies, which might introduce a bias. ② Some studies did not fully report the methods of allocation concealment. ③ Most included patients have come from China, which limits the conclusion to be applied to the wider population. ④ Due to the need for treatment, some studies did not utilize the blind method, which may have some limitations. ⑤ Some RCTs in this review have short follow-up time and lack long-term follow-up for efficacy and survival evaluation. ⑥ Target temperature, TH duration, rewarming rate, and other treatment indicators are different in the included studies. Only a few RCTs have available inflammatory index data, and there are few patient data, such as TNF-*α* and IL-1*β* levels. Therefore, the results of this part of data should be treated with caution.

### 4.3. Perspectives

#### 4.3.1. Inspiration for Future Research

Thrombolysis combined with mild hypothermia is a safe and effective intervention for ACI. However, this does not rule out the possibility that some experiments only report efficacy but neglect adverse reactions. Therefore, the safety of combination therapy needs to be further verified. We recommend that researchers should conduct well-designed clinical trials, standardize uniform diagnostic, treatment, efficacy, and adverse reactions criteria, and further examine the drug effects, mechanisms, and safety of ACI treatment in future studies. Additionally, thrombolysis combined with mild hypothermia has not been widely used for treating ACI. Therefore, these limitations lead to insufficient evidence, and more high-quality RCTs are needed.

## 5. Conclusion

Thrombolysis combined with mild hypothermia presents advantages in treating ACI than thrombolysis alone and exhibits a higher therapeutic effective rate, less oxidative stress as shown by lower SOD and higher GSH-Px, and lower NIHSS, MDA, and IL-1*β* than thrombolysis alone. However, due to the insufficient reports and the limited quality of available studies, the conclusion needs to be applied carefully.

## Figures and Tables

**Table 1:** Details of the 26 RCTs.

Study (published year)	Study type	Country	Sex (M/F)	Females (%)	Age (years) (mean ± SD)	Duration (hours)	Initial NIHSS	Follow-up (days)
Bi (2011) [23]	RCT	China	T: 10/21	T: 32.3	T: 68.45 ± 6.89	T: 2.5 ± 1	T: 11.4 ± 2.8	90
			C: 13/18	C: 58.1	C: 68.55 ± 6.28	C: 2.5 ± 1	C: 11.0 ± 2.7	

Patrick (2017) [24]	RCT	America	T: 34/29	T: 46	T: 65.5 ± 10.3	T: 2.0 ± 0.5	T: 14.1 ± 4.8	90
			C: 35/22	C: 39	C: 67.5 ± 11.1	C: 2.15 ± 0.5	C: 14.5 ± 4.9	

Katja (2014) [25]	RCT	Australia	T: 12/6	T: 33	T: 70	—	T: 11	90
			C: 8/10	C: 56	C: 66		C: 14	

Ovesen (2013) [26]	RCT	Denmark Sweden	T: 9/8	T: 47.1	T: 62.3	T: 1.68	T: 8	90
			C: 8/6	C: 42.9	C: 65.9	C: 3.74	C: 9	

De (2004) [27]	RCT	America	T: 13/5	T: 28	T: 60.9 ± 12.1	T: 3.0 ± 0.5	T: 15.2 ± 4.4	37
			C: 6/16	C: 71	C: 67.3 ± 12.5	C: 4.0 ± 0.25	C: 14.6 ± 5.6	

Derk (2001) [28]	RCT	America	T: 5/5	T: 50	T: 71.1 ± 14.3	T: 3.1 ± 1.4	19.86 ± 3.3	90
			C: 5/4	C: 44	C: 68.2 ± 12.3	C: 4.4 ± 1.7		

Hong (2014) [18]	RCT	South Korea	T: 23/16	T: 41	T: 64.5 ± 17.0	<5	T: 16	90
			C: 18/18	C: 64	C: 68.1 ± 13.3		C: 17	

Thomas (2010) [29]	RCT	California	T: 24	45	65.5 ± 12.1	<6	14.0 ± 5.0	90
			C: 24					
H Bart (2019) [17]	RCT	Netherlands	T: 28/21	T: 37	T: 69.6	<6	T: 11	91
			C: 27/22	C: 39	C: 71.1		C: 11	

Cui (2020) [30]	RCT	China	T: 26/15	T: 37	T: 55.16 ± 6.57	2.48 ± 0.60	—	14
			C: 25/16	C: 39	C: 55.29 ± 6.65			

Li (2018) [31]	RCT	China	T: 28	—	62.6 ± 5.1	<4.5	T: 15.75 ± 6.43	14
							C: 16.15 ± 4.06	
			C: 22					

Yang (2020) [32]	RCT	China	T: 23/17	T: 37	T: 64.5 ± 12.3	T: 3.5 ± 0.3	T: 8.1 ± 3.2	7
			C: 25/15	C: 39	C: 62.1 ± 13.9	C: 3.2 ± 0.2	C: 8.2 ± 2.7	

Jin (2017) [33]	RCT	China	T: 45	—	—	—	—	30
			C: 45					

Wang (2018) [34]	RCT	China	T: 28/25	T: 37	T: 51.29 ± 9.14	T: 3.29 ± 0.81	T: 15.47 ± 3.89	—
			C: 29/24	C: 39	C: 52.08 ± 8.62	C: 3.70 ± 0.97	C: 15.83 ± 6.57	

Li (2014) [35]	RCT	China	T: 16/6	T: 37	T: 65.48 ± 7.66	<3	T: 17.4 ± 3.8	30
			C: 14/8	C: 39	C: 63.24 ± 7.74		C: 16.6 ± 6.2	

Wang (2018) [36]	RCT	China	T: 51/49	T: 37	T: 55.2 ± 1.2	T: 1.50 ± 1.10	T: 16.23 ± 1.56	—
			C: 58/42	C: 39	C: 53.7 ± 1.5	C: 1.30 ± 0.60	C: 16.23 ± 1.31	

Sun (2018) [37]	RCT	China	T: 10/15	T: 58	T: 64.25 ± 10.47	T: 3.14 ± 0.55	T: 15.89 ± 7.08	7
			C: 12/13	C: 52	C: 62.14 ± 9.35	C: 3.09 ± 0.71	C: 15.69 ± 6.39	

Pei (2018) [38]	RCT	China	T: 23/17	T: 37	T: 61.11 ± 7.31	T: 3.29 ± 0.81	T: 16.33 ± 5.67	7
			C: 23/17	C: 39	C: 61.47 ± 7.02	C: 3.70 ± 0.97	C: 16.85 ± 5.31	

Hu (2016) [39]	RCT	China	T: 31	—	60.18 ± 10.18	3.56 ± 0.79	T: 15.22 ± 7.57	90
			C: 29				C: 15.74 ± 6.42	

Cai (2020) [40]	RCT	China	T: 60	—	61.3 ± 4.1	3.65 ± 0.64	T: 12.03 ± 2.56	7
			C: 34				C: 12.01 ± 2.45	

Zhao (2018) [41]	RCT	China	T: 18/15	T: 37	T: 60.33 ± 6.29	—	T: 14.78 ± 3.01	45
			C: 17/16	C: 39	C: 61.24 ± 6.44		C: 14.23 ± 2.28	

Wei (2020) [42]	RCT	China	T: 27/21	T: 37	T: 64.5 ± 9.4	T: 2.80 ± 1.00	T: 14.2 ± 3.7	28
			C: 30/19	C: 39	C: 63.8 ± 10.2	C: 2.70 ± 1.20	C: 13.9 ± 3.0	

Yang (2020) [43]	RCT	China	T: 35/33	T: 37	T: 62.43 ± 12.06	<4.5	—	—
			C: 36/32	C: 39	C: 62.75 ± 11.82			

Ma (2017) [44]	RCT	China	T: 8/7	T: 37	T: 66.33 ± 9.81	T: 4.66 ± 0.54	—	—
			C: 7/8	C: 39	C: 68.26 ± 9.53	C: 4.81 ± 0.35		

Zhu (2019) [45]	RCT	China	T: 19/14	T: 37	T: 64 ± 13	<4.5	T: 23.7 ± 2.2	—
			C: 17/16	C: 39	C: 62 ± 13		C: 24.3 ± 3.2	

Liu (2019) [46]	RCT	China	T: 28/38	T: 37	T: 64.26 ± 10.46	T: 3.08 ± 0.72	—	—
			C: 32/34	C: 39	C: 62.13 ± 9.36	C: 3.13 ± 0.56		

Zhang (2021) [47]	RCT	China	T: 25/16	T: 39	T: 59.6 ± 3.2	T: 3.47 ± 0.5	T: 12.01 ± 2.65	7
			C: 25/13	C: 34	C: 60.5 ± 3.3	C: 3.52 ± 0.48	C: 12.02 ± 2.47	

T, treatment group; C, control group; Age and initial NIHSS shown as median values. “—” indicates data not specified RCT randomized controlled trial, NIHSS National Institutes of Health Stroke Scale.

**Table 2:** Cooling characteristics of included studies.

Study (published year)	From ACI (h)	Drugs	Type of TH	Target temperature (C)	Duration of TH (h)	Rewarming done	Rewarming rate (C/h)	Duration of Dewarming (h)	Outcomes
Bi (2011) [23]	6	rtPA	Systemic surface	—	24	Yes	—	—	② ⑧
Patrick (2017) [24]	3	rtPA	Systemic surface	33	24	Yes	0.5/1	12	② ⑧
Katja (2014) [25]	6	rtPA	Systemic surface	35	12	Yes	0.2/1	7	⑧
Ovesen (2013) [26]	12.24	rtPA	Endovascular cooling	33	24	Yes	0.25–0.5/1	—	② ⑧
De (2004) [27]	8.6	rtPA	Endovascular cooling	33	24	Yes	0.2/1	—	② ⑧
Derk (2001) [28]	6.2 ± 1.3	rtPA	Systemic surface	32	47.4 ± 20	Yes	0.21/1	23	⑧
Hong (2014) [18]	<5.5	rtPA	Systemic combined	34.5	48	Yes	0.5/12	48	⑧
Thomas (2010) [29]	<5	rtPA	Systemic surface	33	24	Yes	0.30/1	12	⑧
Bart (2019) [17]	—	rtPA	Systemic surface	34–35	18	Yes	0.2 ± 0.1/1	—	⑧
Cui (2020) [30]	2.84	rtPA	Systemic surface	33–35	24	Yes	0.25/1	12–20	① ⑥ ⑦ ⑧
Li (2018) [31]	<4.5	rtPA	Systemic surface	33–35	24	Yes	0.5/1	12–20	① ② ④ ⑤ ⑥ ⑦ ⑧
Yang (2020) [32]	<6	rtPA	Systemic surface	33	—	Yes	0.3/1	12	② ④ ⑤
Jin (2017) [33]	—	rtPA	Systemic surface	33–35	48–72	Yes	<0.1	24–28	① ⑧
Wang (2018) [34]	<6	rtPA	Systemic surface	34	24	Yes	0.25/1	—	① ② ⑧
Li (2014) [35]	3	rtPA	Endovascular cooling	33	24	Yes	0.3	12	② ⑧
Wang (2018) [36]	<6	rtPA	Systemic surface	32–35	24	Yes	0.25	—	② ④ ⑤
Sun (2018) [37]	3.14	rtPA	Systemic surface	33–35	24	Yes	0.2	12–24	① ② ④ ⑤
Pei (2018) [38]	—	rtPA	Systemic surface	33–35	24	Yes	0.25	—	① ② ④ ⑤ ⑧
Hu (2016) [39]	—	rtPA	Systemic surface	33–35	24	Yes	0.15–0.25	12–20	① ② ④ ⑤ ⑧
Cai (2020) [40]	3	rtPA	Systemic surface	33–35	24	Yes	0.5	12–20	② ④ ⑤ ⑧
Zhao (2018) [41]	—	rtPA	Systemic surface	33–35	48–72	Yes	0.1	24–48	① ② ⑧
Wei (2020) [42]	—	rtPA	Systemic surface	33–35	48–72	Yes	0.1	24–48	② ③ ⑤ ⑥ ⑧
Yang (2020) [43]	—	rtPA	Systemic surface	33–35	24	—	—	—	① ③ ④ ⑤ ⑧
Ma (2017) [44]	4.66	rtPA	Systemic surface	33–35	24	Yea	0.15	48	②
Zhu (2019) [45]	—	rtPA	Systemic surface	33–35	72	Yes	0.1	24–48	① ② ③ ④ ⑤ ⑥ ⑦
Liu (2019) [46]	<5	rtPA	Systemic surface	34–35	24	Yes	0.25	—	① ② ④ ⑤
Zhang (2021) [47]	0.5–3	rtPA	Endovascular cooling	33–35	24	Yes	0.5	12–20	② ④ ⑤ ⑧

T, treatment group; C, control group. Outcomes: ① clinical efficacy; ② NIHSS; ③ GSH-Px; ④ SOD; ⑤ MDA; ⑥ IL-1*β*; ⑦ TNF-*α*; ⑧ adverse drug reaction. “–” indicates data not specified.

## Data Availability

The data used to support the findings of this study are included within the article.
